# Sensory Organ Investment Varies with Body Size and Sex in the Butterfly *Pieris napi*

**DOI:** 10.3390/insects12121064

**Published:** 2021-11-27

**Authors:** Zahra Moradinour, Christer Wiklund, Vun Wen Jie, Carlos E. Restrepo, Karl Gotthard, Arttu Miettinen, Craig D. Perl, Emily Baird

**Affiliations:** 1 Department of Zoology, Stockholm University, 106 91 Stockholm, Sweden; christer.wiklund@zoologi.su.se (C.W.); vun.wen.jie@zoologi.su.se (V.W.J.); ernesto.restrepo@zoologi.su.se (C.E.R.); Karl.Gotthard@zoologi.su.se (K.G.); cperl1@asu.edu (C.D.P.); emily.baird@zoologi.su.se (E.B.); 2 Swiss Light Source, Paul Scherrer Institute, 5234 Villigen, Switzerland; arttu.i.miettinen@jyu.fi; 3 Department of Physics, University of Jyvaskyla, 40014 Jyvaskyla, Finland; 4 Department of Biology, Lund University, 223 62 Lund, Sweden; 5 School of Life Sciences, Arizona State University, Tempe, AZ 85281, USA

**Keywords:** *Pieris napi*, eye, antenna, wing, proboscis, allometry, sensory system, body size

## Abstract

**Simple Summary:**

Pollinating insects rely on a range of senses such as vision, olfaction, gustation, and mechanosensation to utilise, locate, and fly between floral resources. The size of different sensory organs determines their sensitivity and provides an indication of their relative importance—larger organs can enhance sensitivity by increasing the number or size of sensing structures. However, increasing the relative size of an organ would require additional energy for developing and maintaining it. This likely leads to a trade-off between the energy invested into different sensory systems within individuals. To explore how the size of the sensory organs vary with body size in insect pollinators and how the energetic investment is divided, we performed a series of morphological measurements on the eyes, antennae, proboscis, and wings of male and female *Pieris napi* butterflies with a range of body sizes. We found that only antenna (in females) and wing size (in males and females) increased with body size. Males also had larger eyes and antennae compared to females regardless of body size. Our results provide insights into how the sensory morphology of these butterflies varies with body size and sex, and highlights unusual instances of organs that do not scale with body size.

**Abstract:**

In solitary insect pollinators such as butterflies, sensory systems must be adapted for multiple tasks, including nectar foraging, mate-finding, and locating host-plants. As a result, the energetic investments between sensory organs can vary at the intraspecific level and even among sexes. To date, little is known about how these investments are distributed between sensory systems and how it varies among individuals of different sex. We performed a comprehensive allometric study on males and females of the butterfly *Pieris napi* where we measured the sizes and other parameters of sensory traits including eyes, antennae, proboscis, and wings. Our findings show that among all the sensory traits measured, only antenna and wing size have an allometric relationship with body size and that the energetic investment in different sensory systems varies between males and females. Moreover, males had absolutely larger antennae and eyes, indicating that they invest more energy in these organs than females of the same body size. Overall, the findings of this study reveal that the size of sensory traits in *P. napi* are not necessarily related to body size and raises questions about other factors that drive sensory trait investment in this species and in other insect pollinators in general.

## 1. Introduction

To efficiently utilise, locate, and fly between floral food rewards, pollinating insects rely on a range of senses, including vision, olfaction, gustation, and mechanosensation. The compound eyes perceive relevant information across the span of light intensities that typically occur during the species’ activity period and are tuned to detecting flowers of a particular size and colour range. The antennae have sensory structures that detect not only olfactory cues such scents and pheromones, but also temperature and mechanosensory cues [1,2,3,4]. The proboscis in this group of pollinators is used for finding and feeding on nectar [5,6,7] as well as for determining corolla depth and sensing the chemical composition of nectar [8,9,10]. The wings, which are primarily tasked with generating lift during flight and regulating heat during basking [11], have mechanosensory structures that detect airborne vibrations [12,13,14].

Along with finding food, mating and reproduction are also central to driving the shape and size of sensory organs in insect pollinators. Males and females within a species often exhibit sex-specific specialisations—or sexual dimorphism—in their sensory systems. In pollinating butterflies for instance, females usually invest in sensory traits that facilitate detecting host plants for oviposition, such as vision and olfaction [15,16], whereas male sensory traits are mostly optimised for locating females [17,18]. Males of some butterfly and hoverfly species have relatively larger eyes, larger facets, and a higher facet density for a given body size, which helps them detect and intercept females [19,20,21]. In the silk moth *Bombyx mori* and the giant silk moth *Antheraea polyphemus*, males have enlarged antennal structures that increase their olfactory sensitivity to successfully track the females’ sex pheromone [22]. These sex-specific variations in sensory traits reflect differences in behavioural (mating, oviposition, predator avoidance, conspecific communication, competition, and foraging activity) and physiological (feeding and energy intake, energy expenditure, and fecundity or sperm production) requirements [18].

The relative size of a sensory organ with respect to body size is typically related to its importance for the animal, as larger organs can help to improve sensitivity and/or discrimination of relevant signals but require a greater energetic investment. Sensory systems are energetically costly both to build and to maintain, so the extent to which different organs can grow relative to body size must be traded off against one another [23]. Such trade-offs generate diversity in the relative size of sensory traits both within and between species and will ultimately determine how an individual can respond to its environment and changes within it [24] across its lifespan.

Due primarily to anthropogenic activities, the habitats of pollinating insects are currently undergoing a multitude of rapid changes that will severely modify the environmental cues that their sensory systems have been optimised to detect. To better understand how these insects will respond to these new changes, we need a more comprehensive understanding of sensory trait investment both within and between species. To date, studies on sensory trait investment in insect pollinators focus either on the eyes [19,25,26], or a combination of the eyes and antennae [27,28,29] or on the proboscis [5,30,31]. However, comprehensive studies on sensory organ size variations and the energetic investment among these traits in relationship to body size in pollinating insects are limited at both the inter-specific and intra-specific levels.

To begin to address this knowledge gap, we performed a comprehensive allometric study—an analysis of how organ size varies relative to body size—on the sensory systems of males and females of a temperate butterfly species *Pieris napi*. This butterfly is an ecological generalist that is active across multiple habitat types and therefore likely needs to cope with a wide range of sensory cues. In addition, there is a distinct difference in sex-specific behaviour that likely favours different sensory investments: the species is protandrous, leading to early emerging males that patrol for females. Later in the season, females emerge for mating and oviposition [32,33]. We focused our analyses on the allometric scaling relationship of sensory traits mainly related to foraging activity and reproduction—eyes, antennae, proboscis, and wings. To provide a better insight into how the size of these sensory organs affect the number of sensory structures that they express, we also analysed the density of the antennal sensilla and ommatidia in the compound eye. We hypothesise that, as in the many pollinating insects that have been the subject of allometric analyses to date [5,26,30,34], there would be a positive relationship between body size and the studied sensory organs but that the rates at which each trait increases in size with body size vary between sexes to reflect the differences in their behavioural ecology.

## 2. Materials and Methods

### 2.1. Study Animals

Wild *Pieris napi* (Lepidoptera: Pieridae) butterflies were collected in late August 2019 in Ransvik, southern Sweden (56°17′3″ N, 12°29′50″ E) and transferred to a laboratory at Stockholm University, where they were kept in butterfly net cages 0.8 m × 0.8 m × 0.5 m in size for breeding. The cages were illuminated by 400 W HQIL lamps between 9 am and 5 pm (8 h photophase). The temperature was 28 °C during the photophase and 20 °C during the scotophase. The butterflies were provided with *Kalanchoe* sp. flowers with 20% sugar solution droplets for feeding and *Alliaria petiolata* leaves for egg-laying.

**Figure A1:**
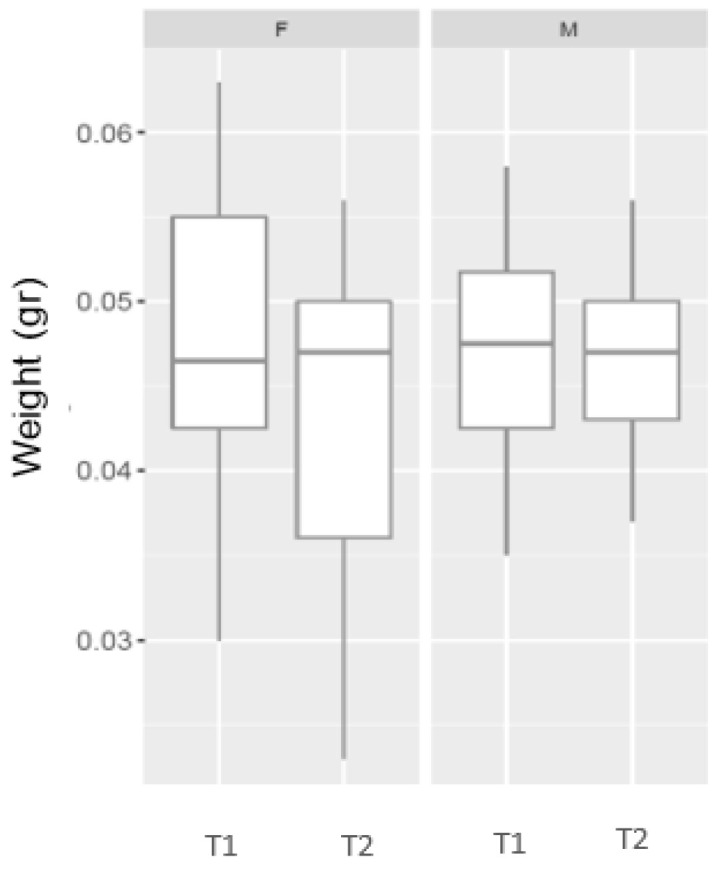
Adult fresh mass between trials (T1 = Trial1, T2 = Trial2) in females and males of *Pieris napi*.

Specimens were collected during two rearing trials in November 2019 and January 2020, and a total of 55 butterflies were used for morphometric measurements. Eggs from the F2 (trial 1) and F4 (trial 2) generations were collected and placed in a rearing room at 23 °C and exposed to 22:2 h L:D cycle and a humidity of 100% until they hatched. Newlyhatched larvae (between day 1 and 2) were transferred to plastic jars, each containing four *Alliaria petiolata* leaves (trial 1) and filled with 15 mm water to maintain humidity for the leaves and the larvae. In trial 2, *Brassica napus* and *Armoracia rusticana* were used for feeding the larvae due to a winter shortage of *Alliaria petiolata*. There was no significance between the weight of adults emerging in Trials 1 and 2 suggesting that the change in host plant did not affect the larval growth (see Figure A1). A total of 120 larvae were placed in the jars and returned to the rearing room until the pupation stage. Larval status was checked daily, and the leaves were kept fresh. Any newly pupated specimens were collected from the plastic jars, weighed on a precision balance (XB 120A Precisa Instruments Ltd., Switzerland, Precisa XB 120A) and sexed under a microscope. These specimens were then transferred to individual plastic 200 mL cups sealed with fabric net and paper on the bottom to facilitate movement after eclosion. Individual cups were transferred to a climate chamber (23 °C: 22 h light, 2 h dark, average humidity: 31%). The chosen lighting regime promotes direct eclosion in *Pieris napi* butterflies from temperate regions. A climate logger (EasyLog, EL-USB-2, Lascar Ltd., Uk) was placed in the chamber and recorded the temperature and humidity at 1 min intervals for 11 days. After seven days, butterflies that eclosed from the chrysalis were transferred to individual plastic jars sealed with fabric net and fed ad libitum with fermented sugar water. These jars were placed in a dark room at 26 °C, 30% humidity for at least 12 h before dissection to ensure full development.

### 2.2. Sample Preparation

Butterflies were euthanised by exposing them to ethyl acetate for 15 min, and their fresh body mass was recorded using a balance (BP 310S, Sartorius) within 5 min of death. The right forewing, left antenna, and proboscis were dissected and, along with the body, mounted on plain paper marked with a 1 × 1 mm black square for scale and photographed. The left antennal club was mounted, and the sulci area was scanned using SEM (HITACHI-TM300, Japan) with 1500× total magnification. In trial 1, the left compound eye was cut from the head and preserved in ethanol (75%). In trial 2, the front part of the head capsule was removed, and the whole head was preserved in ethanol (75%). The eyes and the heads were then stained with 0.5% phosphotungstic acid (PTA), dehydrated in an ethanol series and embedded in epoxy resin according to the methods described in [35]. The eyes and heads were scanned at the TOMCAT beamline (Paul Scherrer Institut, Villigen, Switzerland) with 4× total magnification (voxel size 1.6 µm), with the resulting images being reconstructed using in-house software. See ‘Supplemental methods’ for further information about the preparation procedure.

### 2.3. Morphometric Measurements

Thorax width was calculated using an in-house automated program that isolated the thorax in the image, fitted a bounding box around the body, and took the short axis as the thorax width measurement, which was transformed from pixel to mm values using a 1 × 1 mm reference square. The wing area was measured using an in-house program that isolated the wing in the image, counted the number of pixels it occupied and then converted the occupied area to mm^2^ using the 1 × 1 mm grid paper as a reference. Antennal stalk length, proboscis length, and club area were scaled using the grid paper as a reference and were measured in mm or mm², respectively, in FIJI-image J (64-bit Java 1.8.0_172 [36]). Sensilla density was determined from the SEM images by counting the number of sensilla in a 1 × 1 mm region focused on the sulcus on the third club segment. The reconstructed X-ray scan images containing the left compound eyes were cropped, and the optimal contrast was set in Drishti 2.6.4 image processing software [37]. They were then segmented, and the 3D surface area of each eye was measured in µm^2^ using Amira 6.2.0 (Thermo Fisher Scientific, Waltham, MA, USA). The facet size and facet density were calculated according to the procedures described in [35]. Due to damage incurred during the sample preparation process, it was not possible to measure all sensory organs in all individuals, therefore the sample sizes for each measurement differ slightly. In all cases, the highest possible sample size was considered for analysis.

### 2.4. Data Analysis

We used allometric analyses—that is, analyses of how organ size varies relative to body size—to explore sensory organ investment in *Pieris napi*. Allometric scaling relationships between body size and sensory organ size were explored by applying a log transformation to the data and then fitting following allometric function: log10(Y) = log10(b) + α log10(x), where Y is the size of the organ, b is the initial growth index, α is the scaling exponent (or allometric coefficient), and x is a measure of body size [38]. If α = 1, the organ scales at the same rate as the body (i.e., an individual that is 10% larger in body size will also have organs that are 10% larger), a relationship known as isometric. However, α < 1 describes a negative allometry (hypoallometry), where organ size increases at a slower rate than body size, making it relatively smaller as body size increases. Positive allometry, where α > 1 (hyperallometry), means that organs become relatively larger per unit body size as body size increases [39]. To make a valid comparison with the linear body size measurements, variables expressed in mm^2^ were converted to linear measurements by taking their square root before applying the logarithmic transformation.

All statistical analyses were implemented in R V.4.0.2 (R Core Team 2020. Vienna, Austria) [40]. Allometric slopes were determined for each organ and sex combination using linear regressions in which the size of the trait was fitted as the response variable and body size was fitted as the explanatory variable (Table 1).

Linear mixed models were used to analyse the allometric relationships and to account for variation between sampling trials by using the nlme package [41]. Trial number was included as a random effect, and the sensory organ measurements, body size, and sex were included as fixed effects. The significance of each explanatory variable was assessed using Wald tests (at the 5% level). The facet numbers and facet size were analysed as mean ± standard deviation due to the small sample size. Spearman correlation matrices were computed using the Hmisc and corrplot packages to identify any potential relationships between the sensory organ and body size measurements. This analysis was carried out only using individuals where all such measurements could be taken (female: *n* = 15; male: *n* = 20).

## 3. Results and Discussion

### 3.1. The Relationship between Compound Eye Properties and Body Size

**Figure 1:**
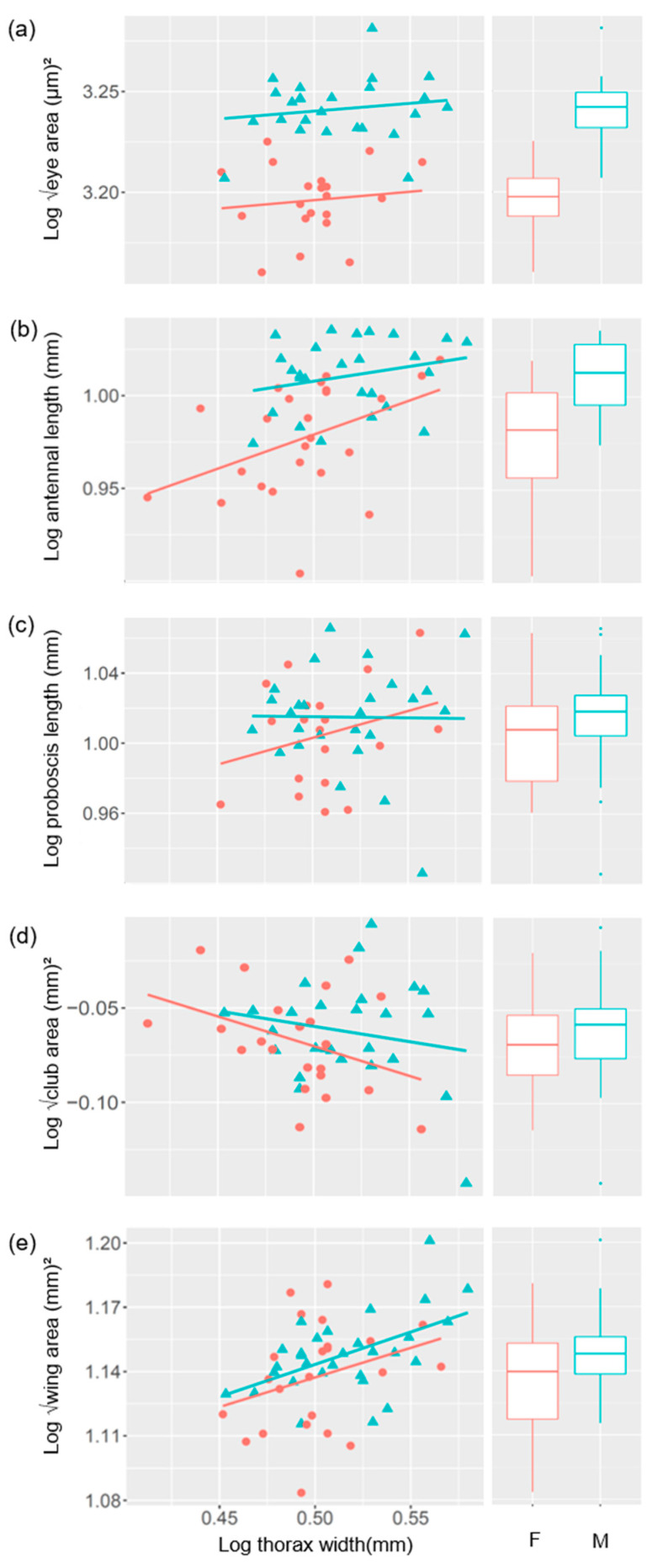
Sensory trait size in *Pieris napi*: allometric scaling relationships and summary box plots of (**a**) eye area, (**b**) antennal length, (**c**) club area, and (**d**) wing area in females (red circles) and (**e**) males (blue triangles). Values on both the x and y axes are log10 transformed.

We found that the surface area of the compound eyes did not increase significantly with body size in *Pieris napi* (t_41_ = 1.7, *p* = 0.1, Figure 1a, Table 1), which means that the eyes of the larger butterflies were proportionally smaller than the eyes of smaller butterflies. This result is surprising as it contrasts with studies on other species of butterflies [19,42] and other insect pollinators, such as bumblebees [34]. One possible explanation is that the eye size of even the smallest *P. napi* is already sufficient for perceiving the visual stimuli they need for feeding and reproduction. If this is the case, then this would allow larger individuals to invest the extra energy resources that are obtained with body size into other organs or in reproductive capacity.

Males and females differed significantly in eye size (t_41_ = 8.7, *p* < 0.001, Figure 1a) and males’ eyes were larger, irrespective of body size. This relationship is also found in other butterfly species such as *Colias eurytheme* and *Eucheira socialis* [43,44]. *P. napi* males actively search for females [33,45], and larger eyes that provide higher sensitivity and acuity [46,47] (by having larger facet sizes or higher facet numbers, respectively) enhance mate finding chance at a distance, thereby mating success. Indeed, in the few specimens where it was possible to analyse the facets of the compound eyes in detail (three males and three females), males had a larger number of facets and a larger average facet area than females (number of facets: 10,327 ± 1072 vs. 8728 ± 935; facet area: 285 ± 13 µm^2^ vs. 279 ± 10 µm^2^; males vs. females, respectively). The sex-specific differences observed in the eyes of *P. napi* reflect well the differences in behaviour, with males seemingly investing in higher sensitivity and resolution, which would make it easier to detect females at a distance. In females, however, higher investment in contrast sensitivity seems to be more prominent, likely due to the need to (improve their ability to) detect flowers and host plants against dark backgrounds. Despite the small sample size in our study, the larger number of facets found in male *P. napi* is consistent with what has been reported in two other species of Pieridae, *Colias erate poliographus*, *P. rapae crucivora* and one Papilionidae, *Papilio xuthus* [19,46]. A larger facet area has been also found in males of *Asterocampa leilia*, (Nymphalidae); although, in this species, facet numbers were higher in females than in males [19].

### 3.2. The Relationship between Antennal Properties and Body Size

We found that antennal stalk length increases with body size in *P. napi* indicating that larger individuals have proportionally longer antennae than smaller individuals (t_46_ = 2.5, *p* = 0.01, Figure 1b). However, the degree to which antennal length scaled with body size was different between males and females (t_46_ = 3.9, *p* < 0.001, Figure 1b, Table 1), with females increasing their antennal length relatively more than males for a given increase in body size. This likely reflects the importance of olfactory sensitivity for females [48], which would be particularly useful for identifying the sex pheromones of males (aphrodisiac pheromone) and the chemical signature of their often visually obscure host plants for oviposition.

**Figure A2:**
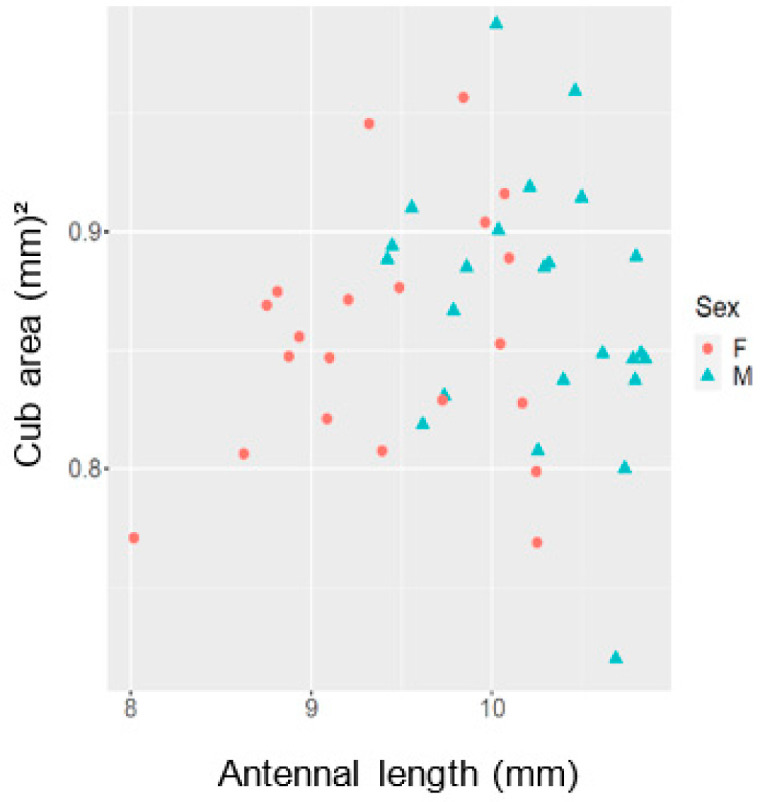
The relationship between club area and antennal stalk length in females (red circles) and males (blue triangles) of *Pieris napi*.

In contrast to antennal length, we found that club area decreases marginally with increasing body size in both sexes (t_44_ = −3.2, *p* = 0.002, Figure 1d, Table 1); although, the variation between individuals was high. As olfactory sensilla density in the club sulci did not vary significantly with club area (t_20_ = −0.3, *p* = 0.7), decreases in club size with body size are likely related to a reduction in olfactory sensitivity. Interestingly, we found no strong correlation between antennal length and club size (t_44_ = −1.5, *p* = 0.1, Figure A2), suggesting that the relative size of these two features of the antennae is not strongly linked. We are not aware of any example in the literature where one part of an organ decreases with increasing body size while another part of the same organ increases. While it is unclear what this unusual relationship means or what the exact function of the club is, our results suggest that it may not be the same as the antennal stalk itself.

### 3.3. The Relationship between Proboscis Length and Body Size

Proboscis length varied greatly between individuals and had no clear relationship with body size (t_42_ = 1.6, *p* = 0.1, Figure 1c) or sex (t_42_ = 0.9, *p* = 0.4, Figure 1c, Table 1). Positive correlations between body size and proboscis length in butterflies and moths have been described previously [5]; although, deviations from this relationship have also been reported. A study on the allometry of nectar feeding butterflies proboscides has shown a positive relationship between proboscis length and body size [31]; although, many species have higher variation in relative proboscis length compared to non-nectar feeders. Relative proboscis length in nectar-feeding butterflies has received less attention at the intra-specific level, and only a few studies have focused on individual variations in butterfly mouthparts [31,49]. The length of the proboscis in butterflies in their natural habitat has been attributed to the shape, size, and corolla depth of the flowers that they feed on [50] as well as to their nectar intake [51]. We therefore propose that the variation in proboscis length in *P. napi* (and potentially also in other butterfly species) may help to minimise competition for floral resources by enabling individuals within a population to feed on different flower types. 

### 3.4. The Relationship between Wing Area and Body Size

There was a significant positive relationship between forewing area and body size (t _49_ = 3.1, *p* = 0.003, Figure 1e), which did not vary between sexes (t_49_ = 1.1, *p* = 0.3, Figure 1e), and had a slope < 1 (Table 1), indicating that it was hypoallometric. Since we measured thorax width as a proxy of body size, the wing-body relationship can be explained by the fact that the size of the thorax is directly related to the muscle mass necessary for supporting the body in—that is, a larger, heavier body requires larger wings and larger thoracic muscles to move them [52,53]. Larger wings could theoretically have higher numbers of mechanosensory bristles sensitive to airborne vibrations along the wing margin due to the increased surface area. These bristles are used to control wingbeats [54] and possibly aid in conspecific communication [12], which can be important for both sexes. However, in a study in a closely-related species *P. rapae* [14], no relationship between wing size and bristle numbers or any difference among sexes was found. It is likely that, although *P. napi* wings contain sensory bristles and glands for sex pheromone, they do not have a direct effect on relative wing size to the same extent as flight performance.

### 3.5. Correlation between Sensory Traits

**Figure 2:**
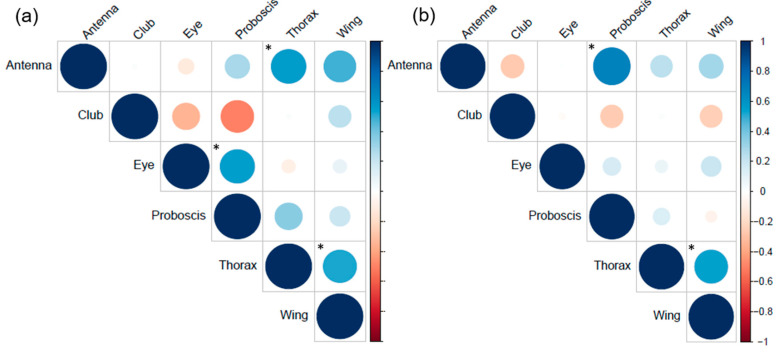
Correlation matrices between the different sensory traits measured in this study and body size in (**a**) females (*n* = 15) and (**b**) males (*n* = 20) of *Pieris napi*. Colours indicate whether the traits increase or decrease in size together (blue and red, respectively). The colour scale indicates the power of the correlation (lighter colours indicating a weak correlation, darker colours indicating a strong correlation) and the size of the circles indicates their level of significance. Correlations that produced significance levels below 0.05 are indicated with *.

To explore not only the relationship between each sensory trait with body size, but also if and how they change size relative to one another, we performed a correlation analysis using specimens from which all sensory organ measurements could be taken. In addition to finding a correlation between antennal length (rs_15_ = 0.57, *p* = 0.03, Figure 2a) and wing size (rs_15_ = 0.52, *p* = 0.04, Figure 2a) to body size in female *P. napi*, we found a significant positive correlation between eye size and proboscis length (rs_15_ = 0.56, *p* = 0.03, Figure 2a). It is possible that these correlations reflect combinations of sensory investments that improve the ability of females to find flowers or host plants; although, further detailed investigation into the sensory cues used for these behaviours is necessary to better understand this. In male *P. napi*, along with the wing-body relationship (rs_20_ = 0.54, *p* = 0.001, Figure 2b), we also found a positive correlation between proboscis length and antennal length (rs_20_ = 0.68, *p* = 0.001, Figure 2b), suggesting that males with longer antennae also have longer proboscides. Over the course of courtship, male butterflies exhibit a form of sexual behaviour waving their proboscis and antennae in front of the female as a form of visual display [55]. Therefore, the correlation between antennal length and proboscis length in males might be an indication of the importance of these sensory organs’ communication for successful mating. Although the function of the correlations between the size of different sensory organs cannot be determined from this study, overall, this analysis reveals that the size of specific sensory organs is highly correlated in butterflies but that these relationships are different in males and females.

## 4. Conclusions

In this study, we explored the allometric relationship between body size and the sensory traits contributing to foraging and reproduction success in males and females of the butterfly *P. napi.* In general, male *P. napi* have larger sensory organs than females on both the absolute and relative scale, which is particularly evident in the antennae and eyes. How this difference in sensory investment between males and females affects their ability to efficiently pollinate and reproduce remains unclear. One possible explanation is that females allocate extra energy gained by increases in body size into non-sensory functions, such as egg production, while males invest in sensory organs that might improve their mating success. The central hypothesis of this study—that insects with larger bodies invest in larger sensory organs to improve sensitivity and/or discrimination of sensory stimuli—was not entirely supported by our data. Of all the traits measured in this study, only wing area (in males and females) and antennal length (in females) increased with body size. This suggests that there is likely to be a strong fitness advantage to increasing the size of these organs as body size increases but that other factors drive variation in eye size, club size and proboscis length. It is possible that the size of these sensory traits can be traded off against one-another. It is unclear whether similar relationships are present in other butterfly or insect species as such comprehensive analyses of sensory organ size within and between individuals are lacking. Further comparative studies on the sensory traits of different species of butterflies from different habitat adaptations and other groups of insect pollinators are necessary to better understand the relationship and interactions of these sensory traits related to their behaviour and habitat specification.

## Figures and Tables

**Table 1:** Allometric slopes of the sensory traits in relation to body size in females and males *Pieris napi*.

Sensory Traits	Sex	Sample Size	Slope	y-Intercept	R^2^
Eye	Female	20	0.084	3.154	−0.041
	Male	25	0.077	3.201	−0.018
Antenna	Female	24	0.367	0.795	0.141
	Male	26	0.159	0.928	0.021
Club	Female	22	−0.315	0.087	0.101
	Male	26	−0.163	0.021	−0.003
Proboscis	Female	19	0.306	0.849	0.022
	Male	27	−0.012	1.021	−0.039
Wing	Female	23	0.276	0.999	0.045
	Male	30	0.302	0.992	0.247

## Data Availability

Upon publication, raw data supporting the findings in this paper will be made available on Figshare (https://doi.org/10.6084/m9.figshare.c.5684827). For review purposes, the data can be accessed through a private link (https://figshare.com/s/bb20d52d4601dd2bf5dd/ accessed on 29 October 2021). The samples used in this study are stored at the Department of Zoology, Stockholm University, Sweden.
